# A198 COLONIC MUC2 MUCIN IS STABILIZED BY FCGBP TO RESIST *SALMONELLA TYPHIMURIUM* INVASION IN GOBLET CELLS

**DOI:** 10.1093/jcag/gwab049.197

**Published:** 2022-02-21

**Authors:** H Gorman, F Moreau, E Beaupre, K Chadee

**Affiliations:** University of Calgary, Calgary, AB, Canada

## Abstract

**Background:**

The colonic mucus bilayer is the first line of innate defense against pathogen invasion in the gut by forming a physical barrier between the lumen and the underlying single layer of mucosal epithelial cells. While MUC2 mucin is the primary component of the mucus layer, it also contains several mucus associated proteins of which FCGBP is of significant interest due to its structural similarities to MUC2 with unknown functions. Here we elucidated the functions of FCGBP in MUC2 mucin in response to *S. typhimurium* ( *St*) adherence and invasion in human colonic goblet cells.

**Aims:**

Hypothesis: FCGBP is coordinately produced with MUC2 mucin and plays an important role in innate host defense. The specific aims are:

1. To determine if FCGBP alters the structural integrity of the mucus layer

2. To determine the role of FCGBP in *St* adherence and invasion of goblet cells

**Methods:**

To investigate whether FCGBP impaired mucus barrier functions, wildtype LS174T (WT) MUC2 mucus-producing goblet cells and LS174T cells with a missense mutation in FCGBP (FCGBP MS) were used. To determine if FCGBP MS led to loss in barrier function, *St* adherence and invasion, and 0.2, 1, and 2 μM fluorescent bead penetration through the mucus monolayers were quantified. MUC2 and FCGBP mRNA and protein expression induced by *St* were analysed by RT-PCR and Western blotting. *St*-induced pro-inflammatory cytokines responses were analyzed by RT-PCR and human focus 15-plex cytokine and chemokine array. Cell killing was enumerated by LDH assay.

**Results:**

FCGBP MS cells exhibited significant loss in MUC2 mucus barrier function as quantified by fluorescent beads penetration closer towards the epithelial cell surface temporally as compared to WT cells independent of bead size. Adherence of *St* was significantly increased in FCGBP MS cells and induced robust MUC2 mRNA and protein expressions in a time-dependent manner. Similarly, *St* elicited robust expression of pro-inflammatory cytokine mRNA and protein release in FCGBP MS as compared to WT cells. FCGBP MS were readily invaded by *St* that resulted in increased cell death as compared to WT cells.

**Conclusions:**

Loss of function by FCGBP MS, but not in WT cells, showed increased penetration of fluorescent beads through the mucus layer that resulted in increased *St* adherence, invasion, and cell death. In WT cells, FCGBP and MUC2 were coordinately upregulated in response to *St*. The concurrent increase in pro-inflammatory cytokine expression in FCGBP MS cells in response to *St* suggests that bacteria directly interacted with the cell surface indicative of an impaired mucus layer. The overall trend for increased bacterial invasion, pro-inflammatory response and cell death in FCGBP MS demonstrates that FCGBP was critical in providing structural integrity and protective functions of the mucus layer.

**Funding Agencies:**

CIHR

